# A Microphysiological Approach to Evaluate Effectors of Intercellular Hedgehog Signaling in Development

**DOI:** 10.3389/fcell.2021.621442

**Published:** 2021-02-09

**Authors:** Brian P. Johnson, Ross A. Vitek, Molly M. Morgan, Dustin M. Fink, Tyler G. Beames, Peter G. Geiger, David J. Beebe, Robert J. Lipinski

**Affiliations:** ^1^Department of Biomedical Engineering, University of Wisconsin, Madison, WI, United States; ^2^Department of Pharmacology and Toxicology, Michigan State University, East Lansing, MI, United States; ^3^Department of Biomedical Engineering, Institute for Quantitative Health Science and Engineering, Michigan State University, East Lansing, MI, United States; ^4^Molecular and Environmental Toxicology Center, University of Wisconsin, Madison, WI, United States; ^5^Department of Comparative Biosciences, School of Veterinary Medicine, University of Wisconsin, Madison, WI, United States

**Keywords:** gene environment interaction, chemical screening, paracrine signaling, cleft lip and palate, embryonic morphogenesis, epithelial mesenchymal cross-talk, 3D extracellular matrix, signaling gradient

## Abstract

Paracrine signaling in the tissue microenvironment is a central mediator of morphogenesis, and modeling this dynamic intercellular activity *in vitro* is critical to understanding normal and abnormal development. For example, Sonic Hedgehog (Shh) signaling is a conserved mechanism involved in multiple developmental processes and strongly linked to human birth defects including orofacial clefts of the lip and palate. SHH ligand produced, processed, and secreted from the epithelial ectoderm is shuttled through the extracellular matrix where it binds mesenchymal receptors, establishing a gradient of transcriptional response that drives orofacial morphogenesis. In humans, complex interactions of genetic predispositions and environmental insults acting on diverse molecular targets are thought to underlie orofacial cleft etiology. Consequently, there is a need for tractable *in vitro* approaches that model this complex cellular and environmental interplay and are sensitive to disruption across the multistep signaling cascade. We developed a microplate-based device that supports an epithelium directly overlaid onto an extracellular matrix-embedded mesenchyme, mimicking the basic tissue architecture of developing orofacial tissues. SHH ligand produced from the epithelium generated a gradient of SHH-driven transcription in the adjacent mesenchyme, recapitulating the gradient of pathway activity observed *in vivo*. Shh pathway activation was antagonized by small molecule inhibitors of epithelial secretory, extracellular matrix transport, and mesenchymal sensing targets, supporting the use of this approach in high-content chemical screening of the complete Shh pathway. Together, these findings demonstrate a novel and practical microphysiological model with broad utility for investigating epithelial-mesenchymal interactions and environmental signaling disruptions in development.

## Introduction

Paracrine signaling factors play key roles in embryonic morphogenesis by establishing complex temporospatial gene expression patterns that drive cell differentiation and tissue outgrowth. The temporally dynamic and multicellular nature of developmental paracrine signaling poses challenges to studying this biology both *in vivo* and *in vitro*. Sonic hedgehog (Shh) is a classic example of an intercellular paracrine signaling pathway that is critically important for normal embryonic and fetal development. For example, Shh activity drives orofacial morphogenesis (Lan and Jiang, 2009; Kurosaka, 2015), while targeted pathway disruption results in orofacial clefts (OFCs) of the lip and palate in animal models (Lipinski et al., 2010; Heyne et al., 2015a). Normal development of the upper lip and palate requires the orchestrated proliferation and fusion of embryonic facial growth centers primarily composed of cranial neural crest-derived mesenchyme overlaid by an epithelial layer (Ferguson, 1988; Jiang et al., 2006). This tissue architecture facilitates epithelium-secreted SHH ligand producing a gradient of pathway activation in the cranial neural crest-derived mesenchyme (Lan and Jiang, 2009; Hu et al., 2015; Kurosaka, 2015).

Most birth defects are thought to be caused by interacting genetic and environmental influences (Beames and Lipinski, 2020). Isolated congenital malformations linked to Shh pathway disruption, including OFCs, holoprosencephaly, and hypospadias, are particularly etiologically complex (Murray, 2002; Carmichael et al., 2012; Krauss and Hong, 2016; Beames and Lipinski, 2020). Among the most common human birth defects, OFCs have been studied extensively, though our understanding of causative factors remains inadequate. Efforts to resolve OFC etiology using genetic approaches have identified dozens of associated risk loci (Leslie and Marazita, 2013), but recognized sequence variants are rarely causative. Furthermore, isolated OFC cases generally do not follow Mendelian inheritance patterns, suggesting an important role for environmental influences in OFC susceptibility (Murray, 2002; Roessler et al., 2003; Graham and Shaw, 2005; Juriloff and Harris, 2008; Lidral et al., 2008; Vieira, 2008). Identifying specific environmental factors that disrupt the signaling pathways that drive orofacial morphogenesis and may contribute to OFC risk is a route to prevention strategies and, therefore, an important focus of investigation.

The Shh signaling pathway is inherently sensitive to disruption by environmental chemicals. We have shown that the natural alkaloid cyclopamine inhibits Shh signaling, decreases mesenchymal proliferation, and prevents tissue outgrowth and fusion, leading to cleft lip and/or palate in mouse models (Heyne et al., 2015a; Everson et al., 2017). Numerous other environmental chemicals have been found to disrupt Shh pathway signaling, including cyclopamine-like dietary alkaloids, natural and synthetic pharmaceuticals, and a common pesticide component (Lipinski et al., 2007; Lipinski and Bushman, 2010; Wang et al., 2012; Everson et al., 2019; Rivera-González et al., 2021). Importantly, Shh signaling is sensitive to disruption by a variety of mechanistically distinct chemicals that affect signal transduction at multiple molecular targets within the signaling cascade. These targets range from secretory ligand modification and paracrine shuttling to downstream sensing and transduction events (Jeong and McMahon, 2002; Lauth et al., 2007; Petrova et al., 2013). However, efforts to identify Shh pathway effectors are limited by the simplicity of traditional *in silico* and *in vitro* assays and the time and cost of complex *in vivo* models. An ideal system would replicate key cellular and molecular interactions that, when disrupted, give rise to most isolated birth defects and be amenable to screening-based approaches to test environmentally relevant drug/chemical libraries (Knudsen et al., 2017).

We present a novel microphysiological model (MPM) that recapitulates developmental epithelial-mesenchymal organization and is suited for chemical screening. To model embryonic facial growth processes *in vitro*, a layer of oral epithelium is overlaid on mesenchymal 3-dimensional (3D) microtissues that are supported by an extracellular matrix (ECM). We show that cellular organization and gradients of SHH-driven signaling of orofacial development are recapitulated in this model and that it is well-equipped to screen for chemicals that modulate various distinct targets across the Shh signaling pathway. This microphysiological system provides a novel approach for identifying environmental influences that contribute to OFC susceptibility and a tractable foundation to examine complex gene-environment interaction.

## Materials and Methods

### Chemicals/Reagents

SHH-N peptide (R&D Systems) and SAG (CAS #2095432-58-7, Selleckchem) were used to exogenously induce Shh pathway activity. The potent Smoothened inhibitors cyclopamine (CAS #4449-51-8) and vismodegib (CAS #879085-55-9) were purchased from LC Laboratories. Additional Shh pathway disruptors assessed include U18666A (CAS #3039-71-2, Tocris), RU-SKI 43 (CAS # 1782573-67-4, Tocris), the anti-SHH monoclonal neutralizing 5E1 antibody (Developmental Studies Hybridoma Bank at the University of Iowa), piperonyl butoxide (CAS #51-03-6, Toronto Research Chemicals), and GANT61 (CAS # 500579-04-4, Tocris). The negative control compound benzo[a]pyrene was purchased from Sigma-Aldrich (CAS #50-32-8). All chemicals were dissolved in DMSO or water.

### Maintenance and Engineering of Cell Lines

The embryonic murine mesenchymal cell line 3T3 Shh-Light2, human fetal oral epithelial (GMSM-K), and mouse cranial neural crest mesenchymal (O9-1) cell lines (Gilchrist et al., 2000; Taipale et al., 2000; Ishii et al., 2012) were used as indicated. The Shh-Light2 variant of the 3T3 cell line expresses a *Gli-*driven firefly luciferase reporter enabling real-time evaluation of the SHH-pathway activation (Taipale et al., 2000). We also used two types of GMSM-K cell lines: a *SHH*-null variant and a variant that stably overexpresses human full-length *SHH* (Fan et al., 2004). In addition, each cell line was engineered for *in situ* visualization; GMSM-K SHH-null cells express RFP and GMSM-K SHH overexpressing cells express GFP. GMSM-K and O9-1 cells were maintained in DMEM with 10% FBS and 1% penicillin-streptomycin and maintained in an incubator at 37°C and 5% CO2. 3T3 Shh-Light2 cells were similarly maintained in media containing the selection agents G418 (0.4 mg/mL, Invivogen, San Diego, CA) and zeocin (0.15 mg/mL, Invivogen).

### Device Design and Construction

Devices were designed and modeled with computer aided design (CAD) modeling software (Solidworks, Dessault Systems, Vélizy-Villacoublay, France). SprutCAM software was used to generate toolpaths, and devices were CNC milled (Tormach Inc., Waunakee, WI, USA) from clear 96-well non-tissue culture-treated plates (Corning Inc., Corning, NY, USA). After milling, each plate was cleaned by sonication for 15 min in 100% isopropyl alcohol. Milled plates were washed with water, dried with compressed air, then heated to 70°C. While plate devices were heating, 0.19 mm thick polystyrene sheets (Goodfellow, Huntingdon, England) were cut just slightly larger than the milled plate devices, sprayed with 70% ethanol, and rinsed with water. Sheets were dried with compressed air and added to the 70°C hot plate. Once the devices and sheets reached temperature, 35 μL of acetonitrile was added to milled bonding ports in the upper left-hand corner of the device to bond the polystyrene sheet to the milled plate. Excess acetonitrile was aspirated from adjacent corners and channels to avoid plate etching. This process was repeated for each of the three remaining corner holes in the milled plate resulting in a bonded seal completely around the device. Bonded devices were cooled at room temperature. Excess polystyrene was trimmed from the outside of the device using a handheld razor blade to complete device construction. Devices were then treated with UV light for 15 min and transferred to a biosafety hood for cell culture. Device design and construction are illustrated in **Figure 2**.

### Seeding of Devices

All experimental cultures were seeded and maintained in DMEM supplemented with 1% FBS and 1% penicillin-streptomycin. In luciferase assays, 2 mM VivoGlo luciferin (Promega, Madison, WI, USA) and 25 mM HEPES were included in the culture media. To improve hyaluronic acid attachment to the device, each device well was filled with 3 μL of polyethyleneimine for 10 min. The wells were then aspirated and filled with 3 μL of glutaraldehyde (GA) for 30 min. Following GA treatment, each device well was washed three times with water. Devices were air dried in a biosafety hood before cells were loaded into device wells. While devices were air drying, hyaluronic acid was prepared according to the manufacturer's protocol. Hystem-C, a hyaluronic acid collagen gel solution (Sigma-Aldrich, St. Louis, MO, USA) was mixed 1:1 with a 100,000 mesenchymal/fibroblast cells/μL solution. The hyaluronic acid:cell solution (1.75–3 uL) was added to each device well; therefore, at most 150,000 cells were seeded per well. Microtissues were allowed to polymerize at room temperature for 45 min, then media was added to the top of cultures. One day after mesenchymal seeding, 10 μL of a 4,000 GMSM-K cells/μL solution was loaded into one or both side channels of the well, and, where indicated, 5 μL of cells were also seeded directly on top of the mesenchymal cells to increase signal for screening. 30 min later, wells and channels of the devices were flushed with media to remove unattached cells. A hydraulic head inducing gravity-driven perfusion of the microtissue was created by adding 100–150 μL media to the center well. Perfused media collected in the half moon reservoirs in the bottom well, which were aspirated each day. Media was changed every 1–2 days, and cultures were dosed daily for 3 days or as indicated after seeding.

### Animal Studies

This study was conducted in strict accordance with the recommendations in the Guide for the Care and Use of Laboratory Animals of the National Institutes of Health. The protocol was approved by the University of Wisconsin School of Veterinary Medicine Institutional Animal Care and Use Committee (protocol number G005396). C57BL/6J mice were purchased from The Jackson Laboratory and housed under specific pathogen-free conditions in disposable, ventilated cages (Innovive, San Diego, CA, USA). Rooms were maintained at 22 ± 2°C and 30–70% humidity on a 12-h light, 12-h dark cycle. Mice were fed 2,920× Irradiated Harlan Teklad Global Soy Protein-Free Extruded Rodent Diet (Envigo Teklad Global, Indianapolis, IN, USA) until day of plug, when dams received 2,919 Irradiated Teklad Global 19% Protein Extruded Rodent Diet (Envigo Teklad Global). Mice were set up for timed pregnancies as previously described (Heyne et al., 2015b). Scanning electron microscopy and hematoxylin and eosin (H&E) staining were conducted as previously described (Dunty et al., 2002; Heyne et al., 2015a).

### *In situ* Hybridization (ISH)

ISH analysis was performed as previously described (Heyne et al., 2016) using an established high-throughput technique (Abler et al., 2011). Embryos were processed whole or embedded in 4% agarose gel and cut in 50 μm sections using a vibrating microtome. Embryos were imaged using a MicroPublisher 5.0 camera connected to an Olympus SZX-10 stereomicroscope for whole mount imaging or a Nikon Eclipse E600 microscope for imaging sections. ISH riboprobe primer sequences: *Ptch1*-fwd GACGTGAGGACAGAAGATTG and *Ptch1*-rev + **T7 leader CGATGTTAATACGACTCACTATAGGG**AACTGGGCAGCTATGAAG.

### Evaluation of Gli-Driven Luciferase

For *in situ* quantification of SHH-induced luciferase activity, culture media included 2 mM VivoGlo luciferin (Promega), which is an injectable *in vivo*-grade substrate that is cleaved by luciferase, producing a luminescent signal. Persistent exposure showed no adverse cytotoxic effect nor reduced luminescent signal in response to SHH ligand (data not shown). Luminescence was measured prior to dosing, as well as after the dosing period to enable normalization to any baseline differences in luminescence across replicates. Luminescence was quantified on a Chemicdoc luminescent imager (BioRad, Hercules, CA, USA) or Pherastar plate reader (BMG, Offenburg, Germany), and magnified images of the SHH-mediated gene expression gradient were enabled by placing a 2X dissecting microscope lens (Leica, Wetzlar, Germany) in the optical path between the camera and the plate. Quantification was performed in ImageLab (Biorad) or ImageJ implemented through Fiji (Schindelin et al., 2012). Cytotoxicity was assessed via multiple means including microscopic evaluation of epithelia, recovery of luminescent activity after chemical wash-out, or evaluation of Renilla luciferase activity using a dual-luciferase assay (endpoint only, Promega). Doses that induced cytotoxicity were not included in regression analysis.

### Histopathology

Microtissues were fixed in 10% formalin for 24 h and a razor was used to remove the bonded thin polystyrene sheet of each device so that a sharp probe could be used to extract the microtissues. Mouse tissues were dissected and fixed in 10% formalin for 24 h. Samples were embedded in paraffin then sectioned at 5 μm. Tissues were stained with H&E to identify the cytoplasm and nuclei of cells, then imaged.

### Statistical Analyses

Quantification of SHH-induced bioluminescent signaling was done in ImageLab software (BioRad Inc). For single comparisons, Student's *t*-test was used to identify significant differences in treatment vs. control (*p* < 0.05). For dose-response experiments, data was background subtracted and normalized to the vehicle control (100% activity). A three-parameter non-linear regression curve-fit was generated in Graphpad Prism to determine antagonism/inhibition and IC50 values were determined for each chemical curve fit. Data are representative of at least two independent experiments.

## Results

### Design and Engineering of a Microplate-Based Microphysiological Model

**Figure 1:**
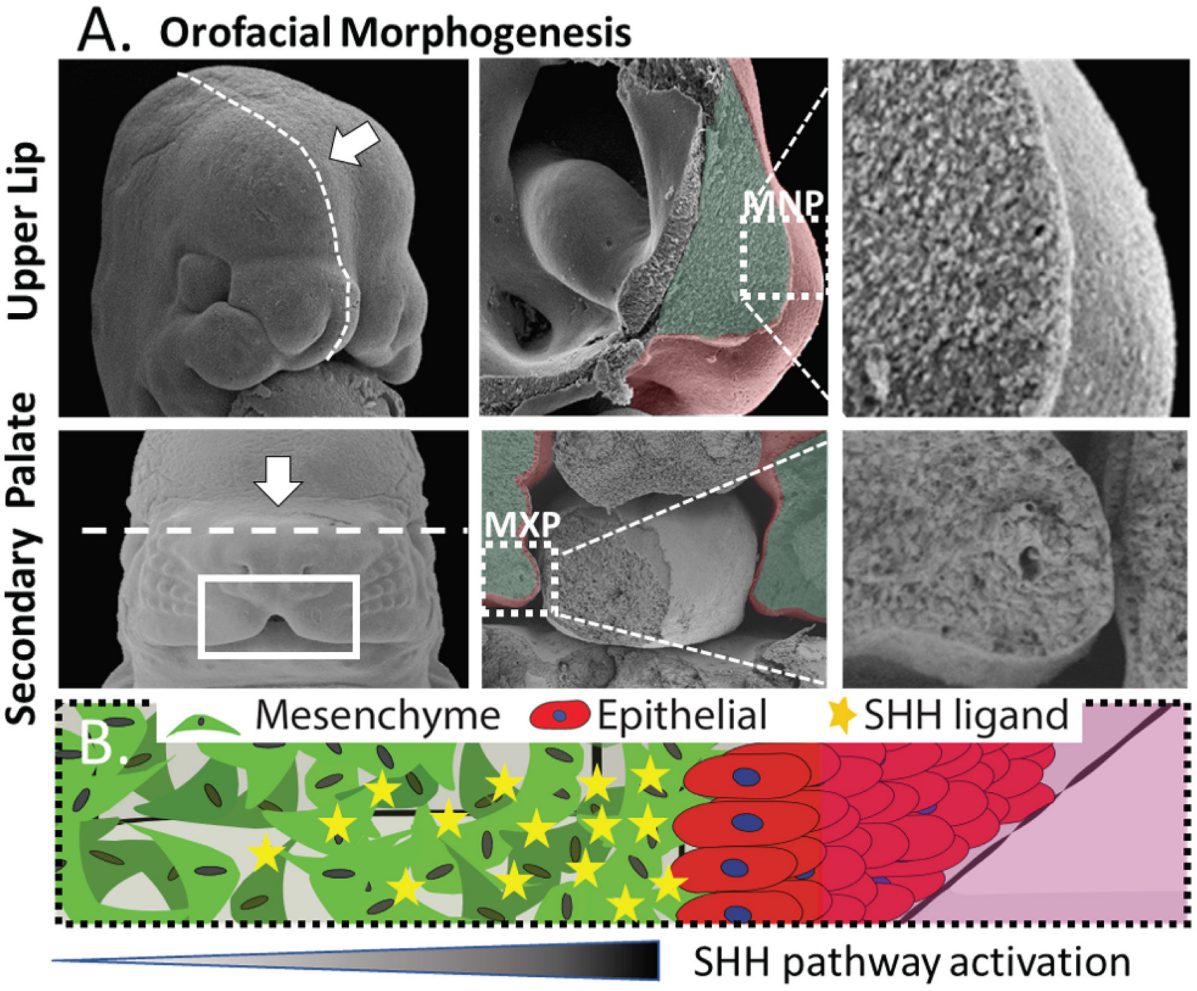
Morphogenesis of the orofacial processes. **(A)** Formation of the upper lip (upper panels) and secondary palate (lower panels) occurs through outgrowth and fusion of embryonic growth centers including the medial nasal process (MNP) and maxillary process (MXP). These tissues consist of a dense 3D mesenchyme covered with ectodermal epithelium. **(B)** Generalized epithelial-mesenchymal tissue architecture of the orofacial processes with high proximal SHH-induced activation.

We sought to create an *in vitro* platform that recapitulates key aspects of epithelial-mesenchymal interaction in development while remaining suitable for drug/chemical screening. The medial nasal process (MNP) and maxillary process (MXP) that form the upper lip and secondary palate, respectively, share morphology as well as intercellular signaling events that orchestrate their development (Figure 1). These structures are composed of a dense 3D cranial neural crest-derived mesenchyme covered by an ectoderm-derived epithelium (Ferguson, 1988; Jiang et al., 2006) (Figure 1A). The tissue outgrowth and fusion of these structures is driven by a continuous epithelial-mesenchymal interaction that is essential for the growth and fusion required to close the upper lip and palate. Deficient outgrowth and/or subsequent fusion of these tissues results in orofacial clefts of the lip and/or palate. Coordinated expansion of these facial growth centers is driven in part by a gradient of epithelium-secreted SHH ligand that induces pathway activation and drives proliferation in the proximal mesenchyme through *Gli*-driven gene transcription (Lan and Jiang, 2009; Hu et al., 2015; Kurosaka, 2015). Both the tissue architecture and epithelial-mesenchymal signaling informed the development of a microtissue design consisting of a dense 3D mesenchyme with an epithelial layer perpendicular to the imaging plane to study epithelial-mesenchymal interactions (depicted in Figure 1B).

### Development of Microtissues That Recapitulate Orofacial Organization in a Throughput-Compatible Format

**Figure 2:**
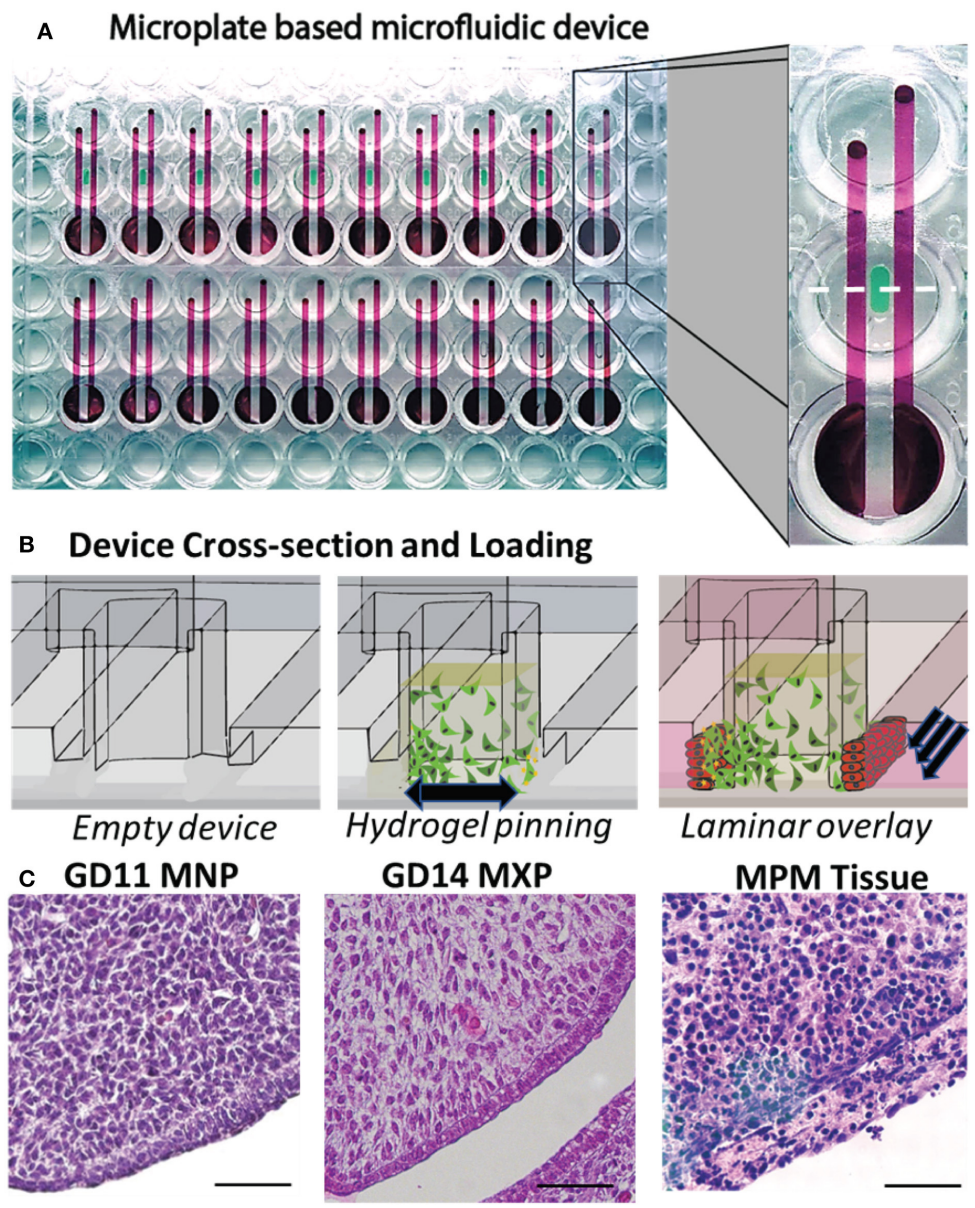
Development of a microplate-based microphysiological culture model. **(A)** Devices incorporate 3 wells of a 96-well plate, where the top and bottom wells are connected through two subsurface microchannels (red) which flank a microtissue well (green) milled into the center well. An array of 20 devices is CNC milled into each plate (bottom view). **(B)** Cross-sectional view of microtissue formation including the empty microtissue well (left panel), addition of the mesenchymal/ECM matrix (center panel), and epithelial overlay (right panel). **(C)** H&E stained formalin-fixed, paraffin-embedded sections of the mouse MNP (left panel) and MXP (center), and O9-1 mesenchymal/GMSM-K epithelial microtissue (right panel), scale bar = 50μm.

To create biomimetic microtissues in a format that is compatible with drug/chemical screening we designed devices using microfluidic principles and manufactured the devices using CNC micromilling of microtiter plates. Devices were milled directly into polystyrene cell culture plates (20 devices/plate) to maintain throughput compatibility, and a thin sheet (190 um) of optically clear polystyrene was bonded to the bottom of the plate to seal the culture chambers (Figure 2A). The devices are manufactured solely from polystyrene to avoid pitfalls of using polydimethylsiloxane (PDMS), which is a common material used for organotypic models and has been previously shown to sequester hydrophobic molecules including many drugs/chemicals (Regehr et al., 2009; Guckenberger et al., 2015). Device design includes a central chamber (Figure 2B, left) that is loaded with a hydrogel-ECM/mesenchymal cell suspension to form the body of the microtissue. Surface tension causes the matrix to pin at 200 μm tall × 1 mm wide openings in the bottom of the chamber (phase barrier) instead of flowing out into adjacent flanking microchannels (Figure 2B, center). The matrix, once polymerized, forms a portion of the wall of the flanking microchannel. Epithelial cells suspended in media are pipetted into the flanking channels. Laminar flow, with a high linear flow rate through the center of the channel and a low linear flow rate at the edges of the channel, leaves cells coating the matrix while cells remaining in the center of the channel are removed via flow (Figure 2B, right). The resulting microtissue consists of a 3D mesenchymal matrix overlaid with epithelial cells perpendicular to the imaging plane. A culture method was developed through many iterations, which resulted in a method whereby a biomimetic mesenchyme was generated by embedding 50,000 cells/μL murine embryonic fibroblast cells (O9-1 or 3T3) in a hyaluronic acid/collagen gel. Hyaluronic acid was used as the microtissue matrix due to its importance in the developing palate (Ferguson, 1988). The microtissues were then overlaid with GMSM-K oral epithelial cells through flanking microchannels to coat the side of the microtissue. To evaluate if the microtissues appear phenotypically similar to the developing orofacial processes *in vivo*, we compared microtissues to the developing lip and palate of embryonic mice at gestational days 11 and 14, respectively. The H&E stains of the murine medial nasal and maxillary processes (Figure 2C, left, center respectively) and the organotypic microtissues (Figure 2C, right) appeared morphologically similar, where the dense mesenchyme was overlaid with epithelium.

### SHH-Induced Gli-Luciferase Enables Exogenous or Endogenous Real-Time *in situ* Quantification

**Figure 3:**
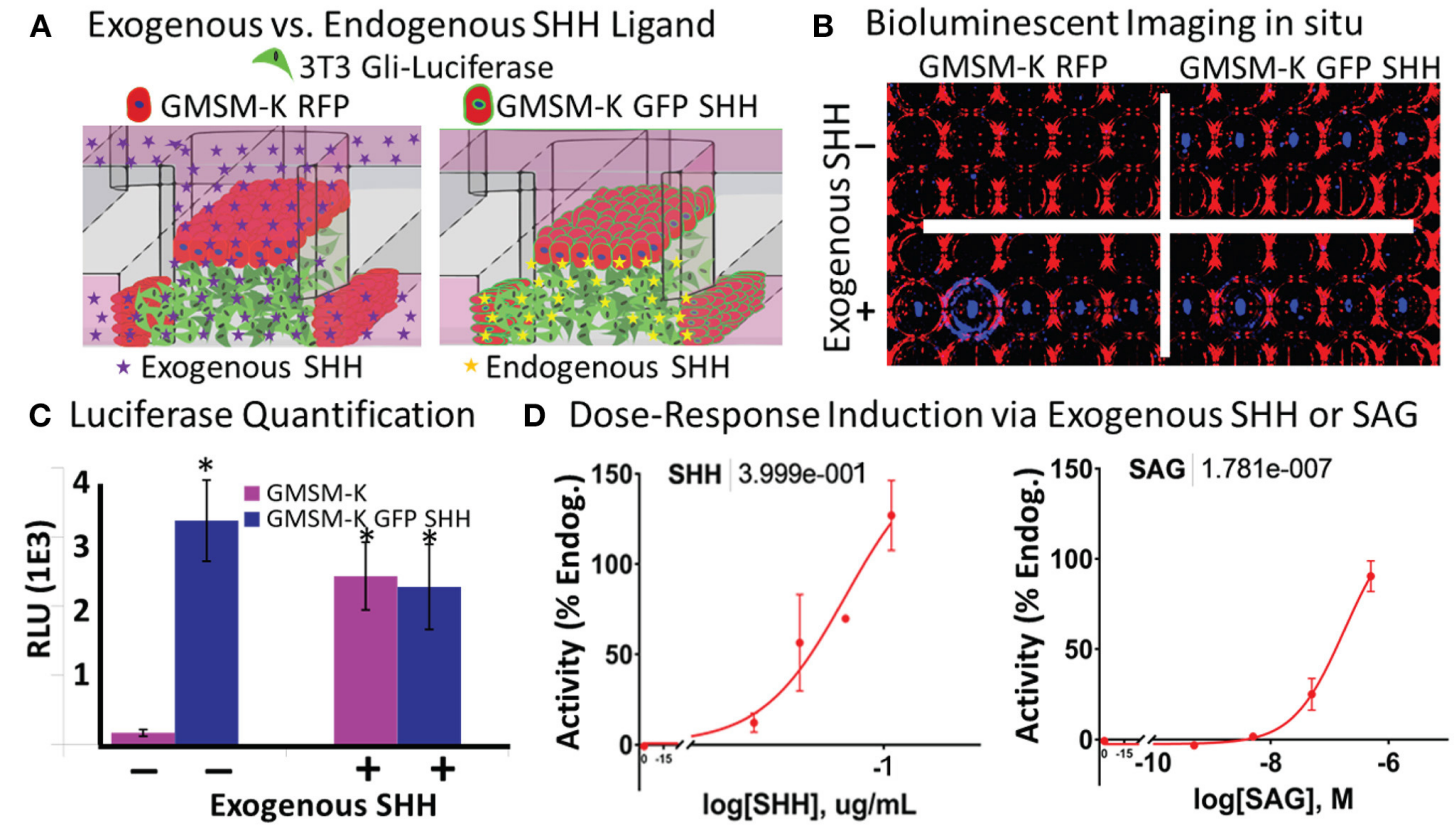
Vital *in situ* quantification of endogenous and exogenous SHH ligand-induced pathway activity. **(A)** Experimental schematic shows incorporation of non-SHH-secreting and SHH-secreting epithelia and exogenously or endogenously derived SHH ligand. **(B)** Brightfield (red) and darkfield (blue) image of bioluminescent signal under different experimental conditions in a single plate. **(C)** Quantification of bioluminescent signal in **(B)** (*p < 0.05 vs. GMSM-K without exogenous SHH, Student's *t*-test). **(D)** Dose-response quantification of exogenously added SHH ligand (left) and SAG (right). Non-linear regression curve fit (Graphpad Prism). EC50 values shown above.

Shh signaling drives the cell proliferation and tissue outgrowth that are critical for orofacial development (Yamada et al., 2005; Kurosaka, 2015; Everson et al., 2017). To enable the real-time evaluation of SHH-driven gene activation, we used a variant of the 3T3 cell line that produces a luminescent signal upon Shh pathway activation. By incorporating a live cell-compatible luciferase substrate into the media, we sought to test if SHH-driven Gli transcriptional activity could be identified from both exogenously added and endogenously secreted SHH ligand *in situ* (Figure 3A). To incorporate endogenous SHH signaling into the microtissues, we applied a variant of the human fetal oral epithelial GMSM-K cells that stably overexpress GFP and SHH (GMSM-K GFP SHH+ cells). The matrix-embedded 3T3 cells were cultured adjacent to GMSM-K GFP SHH+ cells or RFP-overexpressing GMSM-K cells that are SHH- (GMSM-K RFP cells), then exposed to a vehicle control or 0.8 μg/mL of exogenous SHH. After 72 h, microtissues were evaluated for luminescence. 3T3 cells co-cultured with GMSM-K GFP SHH+ cells exhibited a 19-fold higher (*p* < 0.05, Student's *t*-test) signal of *Gli*-driven luciferase compared to 3T3 cells co-cultured with GMSM-K RFP cells. When the GMSM-K GFP SHH+ co-cultures were exposed to exogenous SHH, there was no significant change in *Gli*-driven luciferase activity. In contrast, the GMSM-K RFP co-cultures exhibited a 14-fold higher (*p* < 0.05, Student's *t*-test) signal of *Gli*-driven luciferase when exposed to exogenous SHH (Figures 3B,C). To test dose-responsiveness of the reporter activity, microtissues were exposed to four concentrations of exogenous SHH which produced a concentration-dependent increase in *Gli*-driven luciferase (EC50 = 0.4 μg/mL, non-linear regression curve fit- three parameter) (Figure 3D, left panel). Next, microtissues were exposed to the synthetic Smoothened agonist, SAG, which also showed a concentration-dependent increase in luminescence (EC50 = 178nM, on-linear regression curve fit- three parameter) (Figure 3D, right panel).

### SHH Ligand From the Epithelium Stimulates a Gradient of Pathway Activation in the Mesenchyme

**Figure 4:**
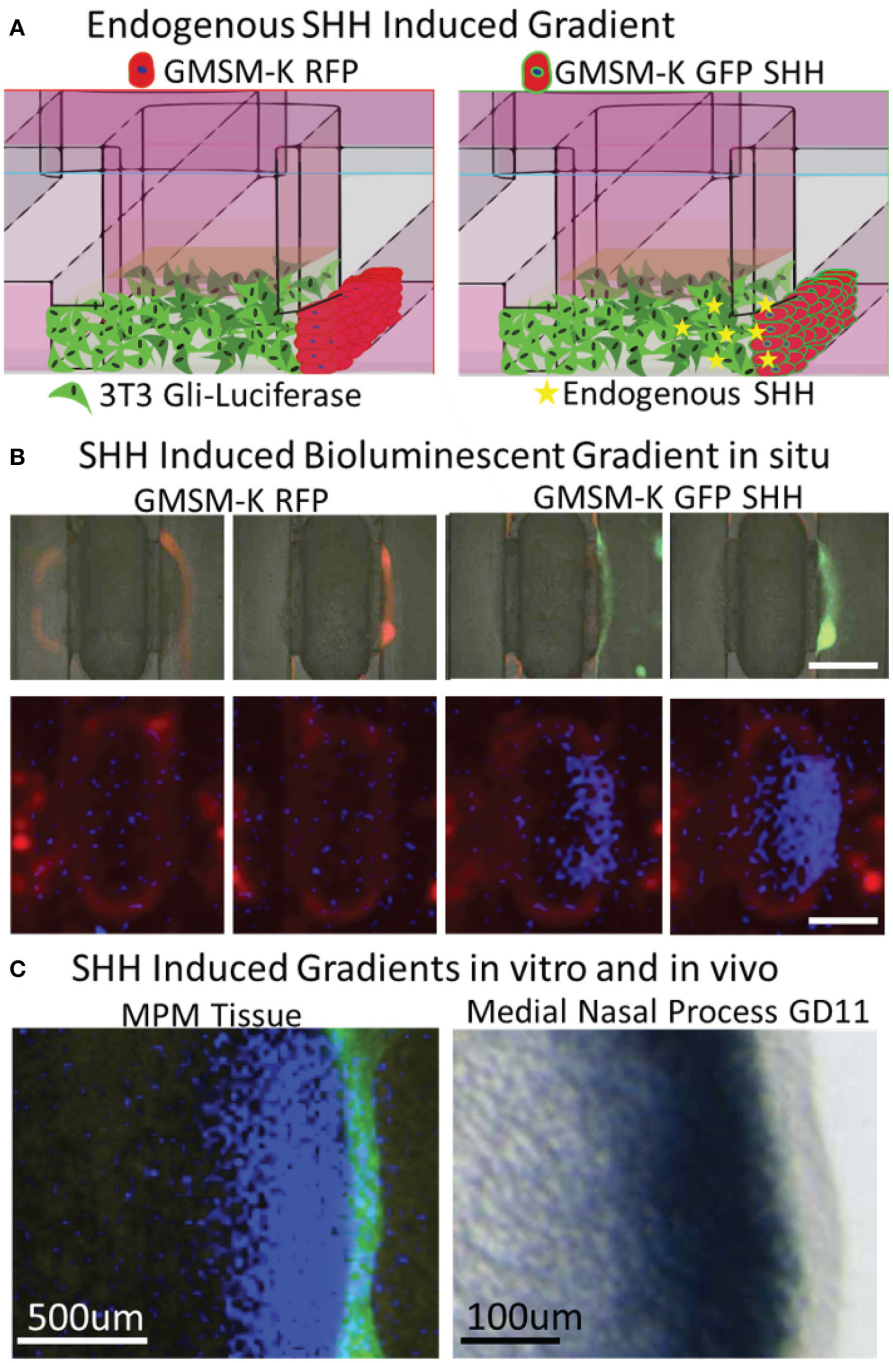
Vital epithelial-mesenchymal SHH gradient *in situ*. **(A)** Experimental schematic shows incorporation of non-SHH-secreting and SHH-secreting epithelia and endogenously produced SHH ligand. **(B)** Brightfield (greyscale) and fluorescent red (530/560 nm) and green (488/515 nm) images indicating GMSM-K overlayed epithelia (upper panels) and bioluminescent signal shows high activity proximal only to SHH secreting epithelia (lower panels), scale bar = 1mm. **(C)** Fluorescent and bioluminescent images taken at higher magnification were integrated to better illustrate gradient (left panel) and compared to ISH staining of SHH-responsive *Ptch1* gene in the medial nasal process of a GD10.25 mouse embryo. Scale bars in luminescent (left) and ISH image (right) are 500 and 100 μm, respectively.

Formation of a gradient of Shh pathway activity is involved in the morphogenesis of many tissues, including the upper lip and palate (Lan and Jiang, 2009; Kurosaka, 2015; Everson et al., 2017) and limbs (Li et al., 2006). To test whether SHH gradients observed during orofacial morphogenesis *in vivo* are recapitulated *in vitro*, microtissues were generated with non-SHH-expressing GMSM-K RFP cells or SHH-expressing GMSM-K GFP SHH+ cells overlaid on one edge of the mesenchyme (one flanking channel) for 48 h (Figure 4A). A merged brightfield and fluorescent image of the microtissues is shown in Figure 4B indicating RFP and GFP expression in the epithelia. Microtissues were then evaluated for *Gli*-driven luciferase using a luminescence imager and showed a gradient of luciferase activity in the mesenchyme with higher activity proximal to the epithelium (Figure 4B). To better compare *in vitro* to *in vivo* gradients, we sought to improve resolution of the luminescent imaging. In a separate experiment, additional optics were added to the light path in the luminescent imager. Using landmarks of the microfluidic device captured by both the luminescent imager and the microscope, we were able to overlay these images at higher resolution for comparison (Figure 4C, right). A photomicrograph of *in situ* hybridization of the SHH-responsive gene *Ptch1* in the MNP of a GD10.25 mouse embryo is provided as an *in vivo* reference (Figure 4C, left). Similar to previous studies (Everson et al., 2017), there was a gradient of SHH-responsive gene expression in the mesenchyme that decreased as distance from the epithelium increased (Figure 4C).

### Sensitivity to Chemical Disruption Across the Shh Signaling Cascade

**Figure 5:**
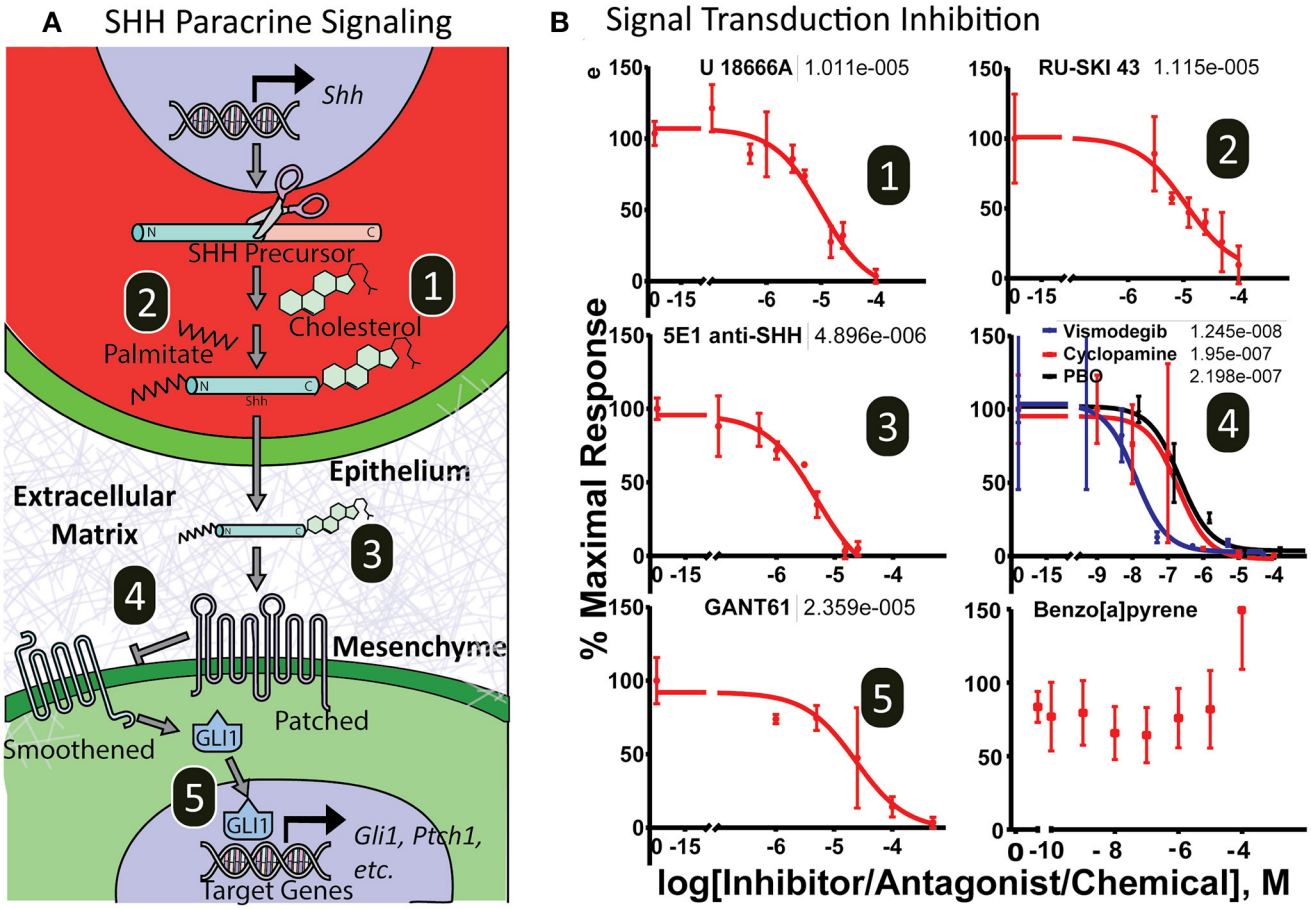
Dose-response curves of SHH pathway inhibitors. **(A)** Illustration of the Sonic hedgehog inter- and intracellular transduction pathway with important molecular targets numbered. **(B)** Microtissues show dose-response inhibition of endogenously derived SHH-induced pathway activity by xenobiotics targeting epithelial secretory SHH ligand cholesterol modification (1) and SHH palmitoylation modification (2); extracellular SHH ligand trafficking (3); and mesenchymal receptor SHH sensing and signal transduction by Patched/Smoothened (4) and Gli-driven transcription (5). The chemical 3-benzo[a]pyrene did not antagonize the pathway. Non-linear regression curve fit (Graphpad Prism). EC50 values shown above.

To test the utility of the platform for screening drug or chemical modulators of the complete Shh signaling cascade, microtissues were exposed to various small molecule inhibitors that target distinct aspects of Shh signal transduction, including epithelial secretion processes (cholesterol modification and palmitoylation), ligand bioavailability, and mesenchymal signal transduction (Smoothened, Gli proteins). Figure 5A illustrates the target of each inhibitor. After 3 days of exposure, cultures were evaluated for luminescence (Figure 5B). Cytotoxicity, monitored by loss of fluorescent signal, washout and luminescent signal recovery, or a lytic cytotoxicity assay, was assessed after dosing, and data from cytotoxic doses were not included in analysis. Inhibitors of SHH posttranslational modification in epithelia affecting SHH secretion include cholesterol (U1886A) and palmitoylation (Ruski-43) inhibitors, which showed IC50 values at 10.1 μM and 11.2 μM, respectively. Importantly, these inhibitors do not exhibit inhibition in a standard 2-dimensional (2D) monoculture assay of 3T3-Gli Luc cells exposed exogenously to SHH ligand (Supplementary Figure 1). The anti-SHH monoclonal 5E1 antibody, which binds and neutralizes secreted SHH ligand, also inhibited *Gli*-driven luciferase signaling in the microtissues with an IC50 of 4.9 μM. Reception of SHH ligand and the subsequent signaling cascade that results in Gli activation in mesenchymal cells can also be inhibited by structurally diverse ligands at multiple molecular targets. The Smoothened antagonists piperonyl butoxide, cyclopamine, and vismodegib inhibited luciferase activity with IC50 values of 219, 195, and 12.5 nM, respectively (non-linear regression curve fit- three parameter). The Gli inhibitor GANT61 also inhibited *Gli*-driven luciferase in a dose-dependent manner with an IC50 value of 23.6 μM (non-linear regression curve fit- three parameter). The chemical benzo[a]pyrene, which can induce cleft palate in rodents through a mechanism independent of Shh signaling, showed no concentration-dependent inhibitory activity. As expected, inhibitors of intracellular Shh signal transduction did exhibit inhibition in a standard 2D monoculture assay of 3T3-Gli Luc cells with IC50 values at or above those seen in the microtissues (non-linear regression curve fit- three parameter) (Supplementary Figure 1).

## Discussion

Here we describe a novel microphysiological culture system that recapitulates key cellular and molecular aspects of developmental Shh paracrine signaling and demonstrate its utility for examining chemical influences that may contribute to birth defects. This elegantly simple microphysiological system mimics both the 3D epithelial-mesenchymal interactions and critical molecular processes of Shh pathway-driven orofacial development, including formation of an *in vivo*-like gradient of pathway activity. Application of a battery of mechanistically diverse chemical inhibitors further demonstrated the sensitivity of the MPM to Shh pathway effectors exhibiting distinct mechanistic targets throughout the inter- and intracellular signal transduction cascade. These observations underscore several important elements of both the developmental fidelity and investigative utility of this microphysiological approach to modeling paracrine signaling in development.

*In vitro* models are often used to elucidate transduction mechanisms and identify xenobiotic pathway modulators, although common culture systems typically fail to recapitulate the complex intercellular signaling pathways that produce morphogen gradients and involve crosstalk between different cell types (Li et al., 2018). Orofacial morphogenesis requires paracrine signaling involving epithelial secretion of SHH ligand and transport through a 3D matrix of SHH-sensing mesenchyme (Lan and Jiang, 2009; Kurosaka, 2015), a paradigm observed in many developmental contexts. 2D cultures can be designed to incorporate a localized population of SHH producers, but the distribution of secreted ligand has been shown to differ between 2D and 3D cultures (Cederquist et al., 2019). The microphysiological approach described here achieves increased cellular complexity by incorporating epithelium and mesenchyme that are predictably engineered in direct contact facilitating analysis. Biologically active SHH ligand secreted from the epithelium induced pathway activity in the adjacent mesenchyme with maximal induction of downstream Shh target genes occurring nearest the epithelial signaling source, mimicking *in vivo* gene expression gradients. Thus, this MPM, along with other recently developed 3D *in vitro* organoid models (Cederquist et al., 2019), may aid in our understanding of how concentration gradients are formed and, more specifically, address persistent questions of how secreted SHH ligand is shuttled through extracellular spaces while remaining available to bind transmembrane receptors to initiate signal transduction (Wierbowski et al., 2020). A novel organoid model consisting of human progenitor/stem cells designed to model disruption of palatal fusion, which occurs later in development was also described recently (Belair et al., 2017). Predictably, a follow-up screen did not detect any effects of the SHH antagonist vismodegib on the viability or fusion of their organoid model (Belair et al., 2018). These models can therefore be viewed as complementary and could be employed in parallel to screen for chemical disruption over a greater range of orofacial development.

The Shh pathway illustrates the importance of designing *in vitro* models that recapitulate the complexity of the inter- and intracellular signaling cascades, rather than just a single point of sensitivity. While Smoothened has been given much attention as a molecular target, the Shh pathway is sensitive to small molecule modulation at several steps. For example, distal cholesterol synthesis inhibitors cause palate and limb malformations consistent with Shh pathway disruption (Chevy et al., 2002), but can fail to impact Shh signaling in simpler *in vitro* cultures (Supplementary Figure 1). Traditional *in vitro* assays examining Shh pathway activity may involve exogenous treatment of a 2D cell monolayer with pre-modified SHH ligand or cells modified to constitutively drive downstream pathway activation (e.g., *Ptch1* knockout or *Gli* overexpression) (Chen et al., 2002; Lipinski and Bushman, 2010), entirely circumventing upstream intercellular signaling events, such as the modification of SHH ligand by cholesterol. Here, application of mechanistically distinct antagonists demonstrated that Shh signaling in the MPM is sensitive to disruption at multiple points in the inter- and intracellular signaling cascade. Shh pathway activity was potently inhibited by blocking cholesterol trafficking (U18666A), SHH palmitoylation (RU-SKI), receptor binding by inactivating secreted SHH ligand (5E1), inhibiting Smoothened (vismodegib, cyclopamine, piperonyl butoxide), and targeting Gli activation (GANT61). Because our engineered approach to *SHH* expression bypasses endogenous *SHH* transcriptional regulators, this approach is unlikely to capture influences that act further upstream, such as those that have been hypothesized for fetal alcohol exposure (Ahlgren et al., 2002; Hong et al., 2020). However, these results demonstrate that the MPM approach described here offers broader sensitivity to mechanistically distinct pathway effectors than typical *in vitro* approaches.

We sought with this MPM to address a significant limitation of microfluidic devices: technical complexity. Even with the appropriate expertise, the technical aspects of advanced systems can contribute to variability between users or prevent widespread dissemination and use (Paguirigan and Beebe, 2008). Machining devices directly into microplates enabled rapid prototyping based on operational feedback and provided a familiar user format without introducing new materials into the experiment. Operationally, engineering devices that leverage the physical properties of fluids at the microscale enabled the simple creation of a microtissue ideally suited to quantify the biology of interest without greatly increasing the expertise required for use. Employing a hydraulic head to facilitate perfusion rather than a mechanical pump allowed us to retain a throughput-compatible format, which is a key advantage for conducting chemical screens, and incorporation of live-cell endpoints greatly reduced handling time and enabled us to monitor activity without sacrificing the culture. Furthermore, the use of polystyrene rather than polydimethylsiloxane (PDMS), a common component of microfluidic devices that sequesters hydrophobic molecules (Regehr et al., 2009), makes this device appropriate for screening a diverse set of compounds.

The operational simplicity of this microphysiological device should enable ready use by other groups for a variety of biological applications in toxicology, pharmacology, and regenerative medicine. The MPM's design affords substantial flexibility and even a “plug and play” paradigm with respect to cells and ECMs, allowing it to model specific developmental environments. For example, although we specifically modeled epithelial-mesenchymal Shh signaling in this study, an iteration of this device was recently used instead to model neurovascular development (Kaushik et al., 2020). Particular attention may be given to Shh-associated congenital malformations, including OFCs, holoprosencephaly, and hypospadias, that are often etiologically complex and difficult to model in *in vitro* systems (Murray, 2002; Carmichael et al., 2012; Krauss and Hong, 2016; Beames and Lipinski, 2020). Full integration of gene-editing techniques such as CRISPR into the MPM could open the door to studying these complex etiologies in a biologically faithful system and even allow for “personalized toxicology” by providing a platform to identify environmental factors that preferentially interact with personal/familial mutations. Another emerging strategy for toxicity testing utilizes *in silico* models built from data collected from *in vitro* and *in vivo* models to predict adverse effects associated with toxicant exposures. A computational model to predict the effects of chemicals on the growth and fusion of the palate was recently reported (Hutson et al., 2017), and informing the development and refinement of *in silico* models and conducting secondary screening of molecules identified by *in silico* screening is another logical niche for MPMs like the one described herein.

The development of model systems that are physiologically relevant but also amenable to mechanistic studies and chemical screening is needed to bridge the gap between existing *in vitro* and *in vivo* models (Beames and Lipinski, 2020). Addressing this need, we present the engineering, construction, and implementation of a novel microphysiological culture model of epithelial-mesenchymal interactions as applied to the Shh signaling pathway. We show that embryonic facial growth processes can be biomimetically modeled *in vitro* by culturing an oral ectodermal monolayer over 3D-embedded mesenchymal cells and that the microtissues and expression patterns phenotypically resemble orofacial morphogenesis. The simplicity of this device makes it adaptable with respect to cell types, pathways, and endpoints of interest. The microplate design also makes this platform amenable to throughput screening, while recent advances in gene-editing technology open the door to its use in investigating gene-environment interactions. Leveraging the physiological relevance and high tractability of this approach illustrate its potential value, particularly for investigating biologically and etiologically complex outcomes including human birth defects like orofacial clefts.

## Data Availability Statement

The original contributions presented in the study are included in the article/Supplementary Material, further inquiries can be directed to the corresponding author.

## Ethics Statement

The animal study was reviewed and approved by University of Wisconsin School of Veterinary Medicine Institutional Animal Care and Use Committee.

## Author Contributions

BJ devised the device design and construction. BJ, RV, and RL planned the experiments. BJ, RV, and PG conducted the experiments. DF prepared specialized cells/reagents. BJ, RV, MM, TB, and RL wrote the manuscript. BJ and MM drew the figures. DB and RL supervised the research and helped develop the idea. All authors contributed to the article and approved the submitted version.

## Conflict of Interest

BJ holds equity in Onexio Biosystems L.L.C. DB holds equity in Bellbrook Labs, L.L.C., Tasso, Inc., Stacks to the Future, L.L.C., Salus Discovery, L.L.C., Lynx Biosciences, Inc., and Onexio Biosystems L.L.C. The remaining authors declare that the research was conducted in the absence of any commercial or financial relationships that could be construed as a potential conflict of interest.
