# Supplementary material for: A Microphysiological Approach to Evaluate Effectors of Intercellular Hedgehog Signaling in Development
Source: Front Cell Dev Biol. 2021 Feb 9;9:621442. doi: 10.3389/fcell.2021.621442 (PMC7900501; doi:10.3389/fcell.2021.621442)
Supplement: Supplementary file 1 [file Data_Sheet_1.docx]

**Supplementary Information**

**Supplementary Methods**

**Seeding of 2D monoculture.** NIH3T3 cells stably expressing Gli-responsive firefly luciferase and TK-renilla luciferase (Shh LIGHTII) were generously provided by Dr Phillip Beachy. Cells were plated in 384 well plates (Falcon, Franklin Lakes, NJ) at 1 × 10^4^ cells/well in 25 μl media. Cells were allowed to attach overnight, and media were replaced with DMEM containing 1% fetal calf serum (FCS) ± recombinant human SHH ligand (0.4μg/mL final concentration), made in a stock solution at 100μg/mL in sterile-filtered 5mg/mL bovine serum albumin (BSA) in phosphate buffered saline (PBS). After media were removed, SHH LightII-3T3 MEF cells were washed once in PBS. Then lysates were collected in Passive Lysis Buffer (Catalog No. E1941; Promega) and luciferase activity was assayed using the Dual-Luciferase® Reporter Assay System (Catalog No. E1910; Promega) according to manufacturer specifications. Relative light units (RLUs) for Firefly and Renilla luciferase were generated using a Enspire multimodal plate reader (PerkinElmer). Data were normalized to positive and negative controls and three-parameter non-linear regression curve fits were calculated using Graphpad Prism. Dose-response data that did not show curve fits are reported as mean +/- standard deviation.

**Supplementary Figures**

**
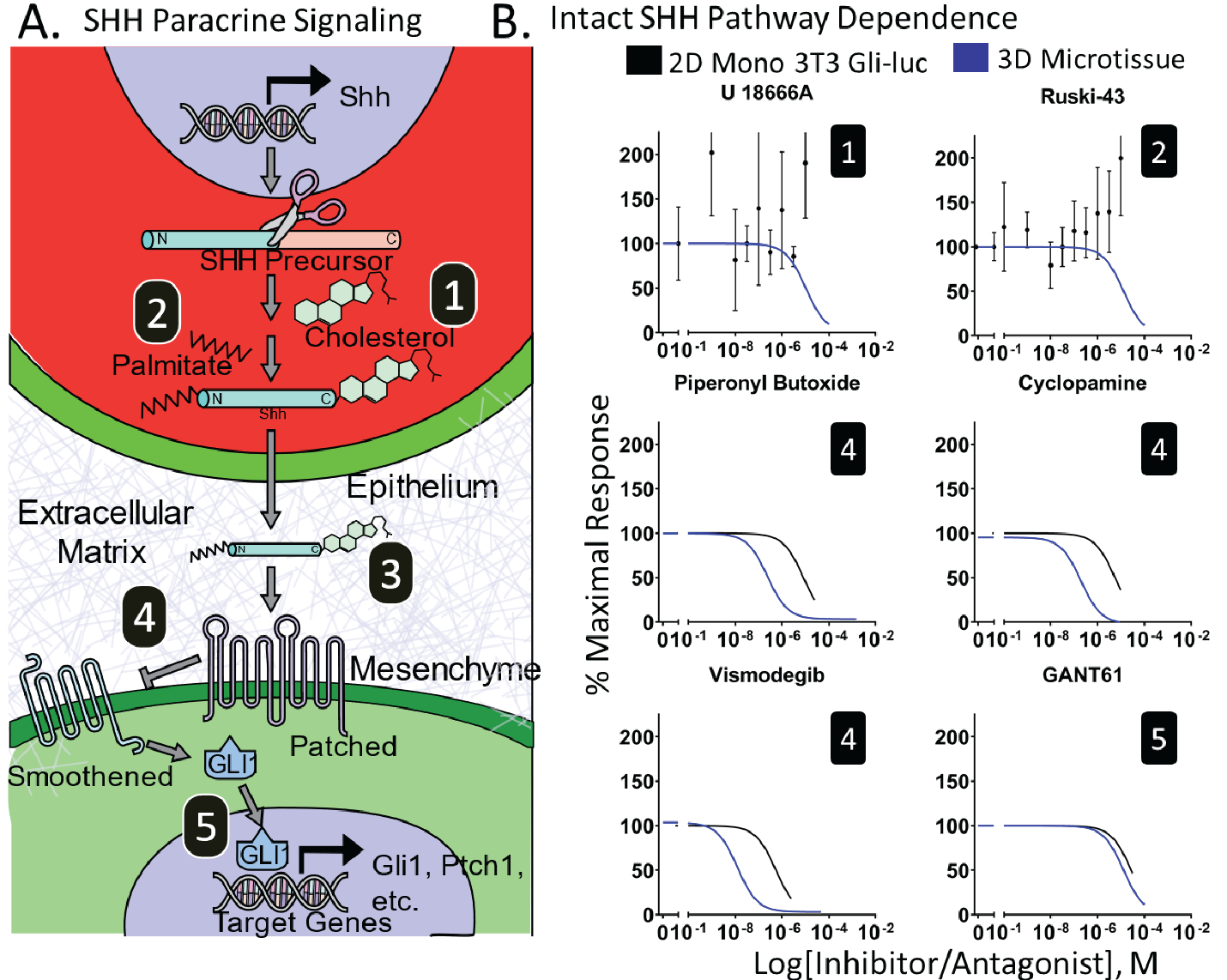
**

**Supplementary Figure 1. Dose-response curves of SHH pathway inhibitors in 2D monoculture and 3D MPM.** A) Illustration of the Sonic hedgehog inter and intracellular pathway transduction with important molecular targets numbered. B) Standard 2D assays (black) do not identify antagonists of SHH secretion including cholesterol modification (1) and SHH palmitoylation modification (2), but do report inhibition of SHH reception including Patched/Smoothened (4) and Gli-driven transcription (5). Non-linear regression curve fits indicate dose-responsiveness (solid lines), means and standard deviation are shown where no dose-response was identified (bar and whisker). Data plotted with 3D MPM data presented in Fig. 5 for comparison (Blue lines).
